# Obituary

**Published:** 1854-09

**Authors:** 


*Obituary.*—Died, of cholera, at Suspension Bridge, Niagara County, on Sunday July 30th, 1854, Eliza, wife of Ryland J. Rogers, M. D. of that place, and daughtor of Robert Pomeroy Esq. of Buffalo.

The brief record, given in this single sentence, leaves untold some circumstances which throw an unusual interest about the death of Mrs. Rogers. Her's was almost the last of the fatal cases of that epidemic. During its continuance Mrs. Rogers had remained at home, refusing to go to a place of safety, and prefering to share her husbands dangers and toils, while, at the same time, she was able to accomplish much good by charitable labors in relieving the sufferings of the wretched poor, who were dying around her.

To all entreaties to remove she returned a calm and cheerful refusal. The beautiful sentiment of Ruth seemed hers; "Entreat me not to leave thee; for whither thou goest, I will go; where thou lodgest, I will lodge; thy people shall be my people, and thy God my God."

With such cheerful devotion, such patient heroism, such christian fortitude, passed from earth to heaven *a true Physician's wife.* H.

